# Apical region correction and global balance: a 3-rods surgical strategy for the treatment of severe and rigid scoliosis

**DOI:** 10.1186/s12891-022-05732-9

**Published:** 2022-08-13

**Authors:** Yang Jiao, Haining Tan, Erwei Feng, Zhen Wang, Youxi Lin, Junduo Zhao, Jianxiong Shen

**Affiliations:** Department of Orthopedics, Peking Union Medical College Hospital, Peking Union Medical College, Chinese Academy of Medical Science, Beijing, People’s Republic of China

**Keywords:** Apical region correction and global balance, Three rods, Severe and rigid scoliosis, Surgical treatment

## Abstract

**Background:**

The treatment of severe and rigid scoliosis is challenging. We developed a surgical strategy for severe and rigid scoliosis since 2014. This study aimed to retrospectively analyze the safety and efficacy of apical region correction and global balance with 3 rods as a surgical strategy for the treatment of severe and rigid scoliosis.

**Methods:**

A retrospective study was performed for patients with severe and rigid scoliosis who underwent one-stage posterior corrective operation using the apical region correction and global balance with 3 rods surgical strategy between February 2014 and April 2020. The inclusion criteria were as follows: [1] Cobb angle > 90°; [2] flexibility < 30%; [3] a minimum 2-year follow-up. Patients were excluded if they had a history of traction or spinal surgery. Coronal and sagittal parameters, including Cobb angle, flexibility, apex vertebra translation, trunk shift (TS), thoracic kyphosis, lumbar lordosis, and sagittal vertical axis (SVA) were measured preoperatively, postoperatively and at the final follow-up. The Scoliosis Research Society 22-item questionnaire was administered preoperatively and at the final follow-up. During the operation, one slightly-bent short rod was placed into the concave side of apical region and correction was achieved by rod-rotation and distraction. Two pre-bent long rods were placed into both sides of the scoliosis and global balance was improved by leveling the proximal thoracic vertebrae and distal lumbar vertebrae.

**Results:**

A total of 41 patients were included, with an average age of 20 years (range, 12–49 years) and follow-up of 34 months (range, 24–58 months). Postoperative correction rate was 53% for scoliosis. There were 14 patients with normal kyphosis before surgery, and 28 patients with normal kyphosis at the last follow-up. 88% of the patients (23/26) with preoperative coronal imbalance (TS > 20 mm) restored coronal balance at the final follow-up. 87% of the patients (14/16) with preoperative sagittal imbalance (SVA > 40 mm) restored sagittal balance at the final follow-up. The mean operation time and blood loss were 286 min and 941 mL, respectively. No patients had neurological complications or implant failure.

**Conclusion:**

The surgical strategy of apical region correction and global balance with 3 rods is a safe and effective alternative for the surgical treatment of severe and rigid scoliosis.

## Background

Severe and rigid scoliosis is a form of scoliosis deformity in which the Cobb angle of the main curve is greater than 90° and flexibility is less than 30% [1]. Severe spinal deformities may lead to dysfunction of the nervous system or cardiopulmonary dysfunction, which in turn affects patients’ growth, appearance, and long-term mortality [2]. The treatment of this condition remains challenging [3–5].

Currently, there are several options for the treatment of severe and rigid scoliosis, including surgical correction after traction [6], anterior release and posterior spinal fusion [7], high grade osteotomy [5], and one-stage posterior spinal correction and fusion [8]. Halo-gravity, halo-pelvic, and skull-femoral traction are usually used to treat severe and rigid scoliosis before or during surgery [9–12]. Although this technique has a certain corrective effect, patients may not tolerate the long treatment cycle, as well as traction-related complications with an incidence of 16% to 28% [5, 12, 13]. Besides, several studies have found that preoperative traction does not increase the final correction rate compared with one-stage spinal fusion. Sponseller et al. compared the correction of severe and rigid scoliosis with and without preoperative halo-gravity traction and found no significant difference in the main coronal curve correction (62% vs. 59%), operation time, and blood loss between the two groups [14]. Anterior release and posterior spinal fusion can also improve the postoperative corrective effect. Zhou et al. used anterior release combined with multiple posterior distraction in the treatment of severe and rigid scoliosis, the correction rates of scoliosis were 58.1% after the anterior release and first posterior corrective surgery and 75.3% after the final corrective surgery [15]. However, multiple operations, pulmonary complications, excessive blood loss, and long hospital stay are the prominent drawbacks of anterior release and posterior spinal fusion, with a complication incidence of 10%–27% [16]. Suk et al. have suggested that for scoliosis with a Cobb angle > 70°, anterior surgery does not significantly affect the correction rate of posterior surgery [17]. Vertebral column resection (VCR) osteotomy and asymmetrical pedicle subtraction osteotomy (PSO) are currently recommended by some surgeons for their good correction rates. However, the incidence of neurological complications and implant failure is high, particularly in patients with intraspinal abnormalities, pulmonary dysfunction, and kyphosis [18–20]. In addition to perioperative complications, the above techniques are associated with long operation time and hospital stay and massive blood loss [15, 21–23].

De Giorgi G et al. used three-rod Cotrel-Dubousset (CD) instrumentation combined with Halo traction or anterior release to correct severe scoliosis in the 1990s, achieving more than 50% correction rate [24]. However, because there were only two pedicle transverse claws at the cranial end, the rod rotation could only be performed in the lumbar area, and the long rod on the concave side had to be turned into two short rods, which were connected by a sliding domino. In addition, because there were only 2–3 hooks in the apical region, the distraction force of the apical region was limited, therefore rigid scoliosis could only be corrected by additional anterior release or traction. With the wide application of pedicle screws, one-stage posterior spinal correction alone can achieve good results in the treatment of severe and rigid scoliosis; however, the incidence of implant failure and pseudoarthrosis has been increasing [4, 23]. In order to reduce surgical complications, shorten the hospital stay and enhance the stability of internal fixation, we have been applied apical region correction and global balance with 3 rods as a surgical strategy for severe and rigid scoliosis since 2014. The purpose of this study was to retrospectively analyze the efficacy and safety of the application of this surgical strategy in the treatment of severe and rigid scoliosis.

## Methods

### Patients

This study was designed as a retrospective analysis of cases in which patients with severe and rigid scoliosis (Cobb angle > 90°, flexibility < 30%) were surgically treated using apical region correction and global balance with 3 rods by the same senior spinal surgeon at one institution between March 2014 and April 2020. Patients were excluded from the study if they had a history of traction or spinal surgery or their follow-up duration was < 2 years.

### Surgical technique

The operation was performed under general intravenous anesthesia, and continuous spinal cord monitor was applied during the operation. The last touching vertebra of the center sacral vertical line (CSVL) on the three-dimensional (3D) reconstruction of the spine CT was usually chosen as the lowest instrumented vertebra (LIV). Partial or full resection of the bilateral facet joints (Schwab grade I or II osteotomy) was performed within the fusion range. Fluoroscopy was used to confirm the location of pedicle screws. The surgical strategy included three steps, as described below:

1. Apical region correction: after the pedicle screws were implanted, one pre-bent short rod was placed on the concave side to connect 3–8 pedicle screws around the apical region. The short rod was placed between the end vertebrae. The purpose of the short rod was to correct the apical region as much as possible without interfering with the placement of the long rod on the concave side. The apical region deformity was corrected by rod rotation and segmental distraction.
2. Global balance: one pre-bent long rod was placed to connect the remaining screws on the concave side and distracted, usually on the outside of the short rod, to restore the global balance based on the Harrington stable zone.
3. Convex side support: a third rod was placed on the convex side and compressed properly to further level the proximal thoracic vertebrae and distal lumbar vertebrae to improve the global balance.

Crosslinks were placed between long rods to control vertebral rotation and improve the stability of internal fixation. The diameter of the rods was 5.5 mm and the material was titanium. Pedicle screws were polyaxial. Hooks were used in some cases where it was difficult to place pedicle screws. Autogenous bone combined with allogeneic bone was used for posterior bone graft fusion.

### Radiographic measurement and clinical evaluation

The coronal and sagittal parameters of the spine were measured before the operation, after the operation, and at the final follow-up. The coronal plane parameters were as follows: (1) Cobb’s angle of the main curve, (2) flexibility of the main curve, (3) apical vertebral translation (AVT), and (4) trunk shift (TS). The sagittal plane parameters were as follows: (1) thoracic kyphosis (TK), (2) lumbar lordosis (LL), (3) pelvic tilt; (4) pelvic incidence, (5) sacral slope, and (6) sagittal vertical axis (SVA). Hypokyphosis was defined as TK < 20°, normal kyphosis was defined as TK within the range of 20° to 45°, and hyperkyphosis was defined as TK > 45° [25, 26].

The operation time, intraoperative blood loss, and implant density were also assessed. The implant density was calculated as follows: total number of implanted screws / (number of fusion segments × 2) × 100%. In addition, all patients completed the Scoliosis Research Society 22-item (SRS-22) outcome questionnaire before the operation and at the final follow-up.

### Statistical methods

IBM SPSS Statistics for Windows, version 25.0 (IBM Corp., Armonk, NY, USA) was used for statistical analysis, and the measurement data were expressed as the mean ± standard deviation. Pair t-test was used to compare the imaging measurement indexes among the three time points (before operation, after operation, and at the final follow-up). A Pearson correlation was conducted to assess normally distributed variables. The significance level was defined as *P* value < 0.05.

## Results

### Patients’ characteristics

Based on the selection criteria, a total of 41 patients (12male and 29 female) with an average age of 20.3 (range, 12–49) years were included in this study. All patients were followed up for an average of 34.1 (range, 24–58) months. The average body mass index (BMI) was 17.86 ± 2.4 kg/m^2^. There were 16 cases of congenital scoliosis, 10 cases of idiopathic scoliosis, 10 cases of neuromuscular scoliosis, 4 cases of Marfan’s syndrome, and 1 case of type I neurofibromatosis (Table 1). The patients with neuromuscular scoliosis were ambulatory.

**Table 1:** Surgical information

Case	Diagnosis	Sex	Age	BMI	Op time (min)	Blood loss (mL)	Apex vertebra	Range of short rod	Fused segments
1	CS	F	16	17.8	320	1125	T8	T4–T12	T1–L3
2	CS	M	16	16.3	310	633	T9	T7–T12	T3–L4
3	CS	M	23	19.0	260	700	T7	T4–T11	T2–L2
4	CS	F	18	15.8	490	1158	T7/L1	T5–T10 T11–L3	T3–L5
5	CS	F	24	17.3	310	1323	T12	T10–L2	T5–L5
6	CS	F	18	19.2	270	578	T10	T6–L1	T3–L3
7	CS	F	12	15.8	300	1050	T8	T4–T12	T2–L3
8	CS	F	13	18.9	280	690	T7	T4–T11	T3–L3
9	CS	M	15	15.6	270	770	T10	T7–T12	T4–L3
10	CS	M	20	21.6	250	2000	T11	T8–L2	T3–L3
11	CS	M	18	22.1	310	1850	T11	T9–L1	T4–L4
12	CS	F	17	15.4	305	900	T9	T7–T11	T3–L3
13	CS	F	21	18.0	220	500	T8/9	T6–T12	T3–L3
14	CS	F	21	15.9	325	750	T11	T9–L1	T3–L5
15	CS	F	15	14.3	290	850	T12	T9–L2	T4–L5
16	CS	F	17	15.2	330	1450	L1	T9–L3	T3–L5
17	IS	F	17	19.1	275	689	T10	T6–L1	T3–L4
18	IS	F	20	20.9	240	722	T9	T6–T12	T2–L3
19	IS	F	49	15.5	250	710	T12	T9–L3	T5–L5
20	IS	F	15	17.9	275	957	T11	T7–L1	T2–L4
21	IS	F	19	22.2	256	484	T10	T7–T12	T4–L4
22	IS	F	41	16.2	220	1058	T9	T8–T11	T3–L3
23	IS	F	36	25.2	275	593	T7	T5–T9	T2–L1
24	IS	F	49	17.1	210	860	T10	T8–T12	T5–L3
25	IS	F	36	17.8	236	755	T10	T8–L1	T4–L4
26	IS	F	19	16.4	230	680	T8	T6–T11	T4–L3
27	NMS	F	18	16.2	320	1050	T7	T5–T10	T3–L5
28	NMS	M	16	16.3	310	755	T12	T10–L2	T5–L5
29	NMS	M	14	15.3	295	950	T10	T8–T11	T3–L3
30	NMS	F	15	22.4	330	1200	T8	T5–T10	T5–L4
31	NMS	M	13	20.2	335	1050	T9	T6–T12	T3–L3
32	NMS	F	14	18.1	270	670	T10	T8–L1	T2–L4
33	NMS	F	15	16.9	375	1500	T12/L1	T10–L2	T2–L5
34	NMS	F	14	17.6	275	930	T9	T7–T12	T3–L4
35	NMS	M	16	16.9	265	848	T11	T9–L2	T3–L4
36	NMS	F	23	21.1	270	615	T10	T6–L1	T3–L4
37	MFS	M	19	15.8	280	760	T9	T5–T12	T3–L4
38	MFS	F	18	18.9	270	975	T8	T4–T11	T3–L3
39	MFS	M	16	17.3	275	390	T8	T6–L1	T2–L3
40	MFS	F	24	16.7	295	1200	T11	T9–L1	T2–L4
41	NFS	M	13	16.2	280	1860	T7	T4–T9	T2–L1

*CS* Congenital scoliosis, *IS* Idiopathic scoliosis, *NMS* Neuromuscular scoliosis, *NFS* Neurofibromatosis scoliosis, *MFS* Marfan syndrome complicated with scoliosis

### Surgical information

The range of the upper fusion vertebrae was T1–T5, and the range of the lower fusion vertebrae was L1–L5. The number of fusion segments ranged from 11 to 16, with an average of 13.6 ± 1.1 segments. The average distance from the cranial end of the concave short rod to the apical vertebra was 2.71 ± 0.8 segments, and the average distance from its caudal end to the apical vertebra was 2.64 ± 0.7 segments. On average, 1.5 crosslinks were used per patient (range, 0–3). The average operation time, blood loss, and implant density were 286.6 ± 50.3 min, 941 ± 341 mL, and 55.6% ± 10.2%, respectively. There was no change in the intraoperative spinal cord monitoring signal during the operation. The full operative details for all patients are presented in Table 1.

### Radiological outcomes

**Fig. 1:**
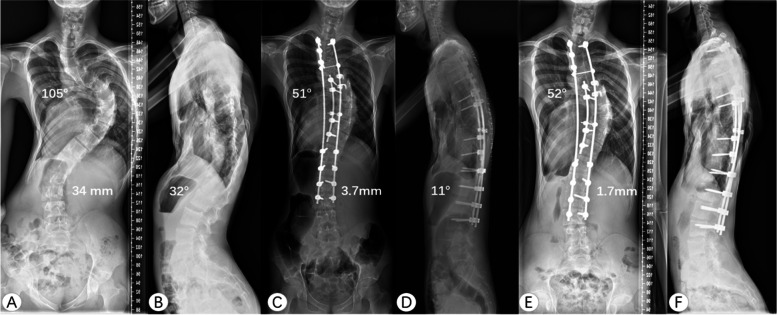
A 16-year-old male patient with severe and rigid scoliosis caused by Marfan syndrome. The preoperative Cobb angle of the main curve was 105° and TS was 34 mm. The thoracolumbar kyphosis was 32° (**A**, **B**). He underwent posterior surgery from T2 to L3 and the apical region correction was from T6 to T11. After the operation, scoliosis and thoracolumbar kyphosis was corrected to 51°, and 11°, respectively. The coronal plane restored balance (TS = 3.7 mm) (**C**, **D**). After two years of follow-up, the correction remains stable and the TS is improved (**E**, **F**). TS, trunk shift

**Fig. 2:**
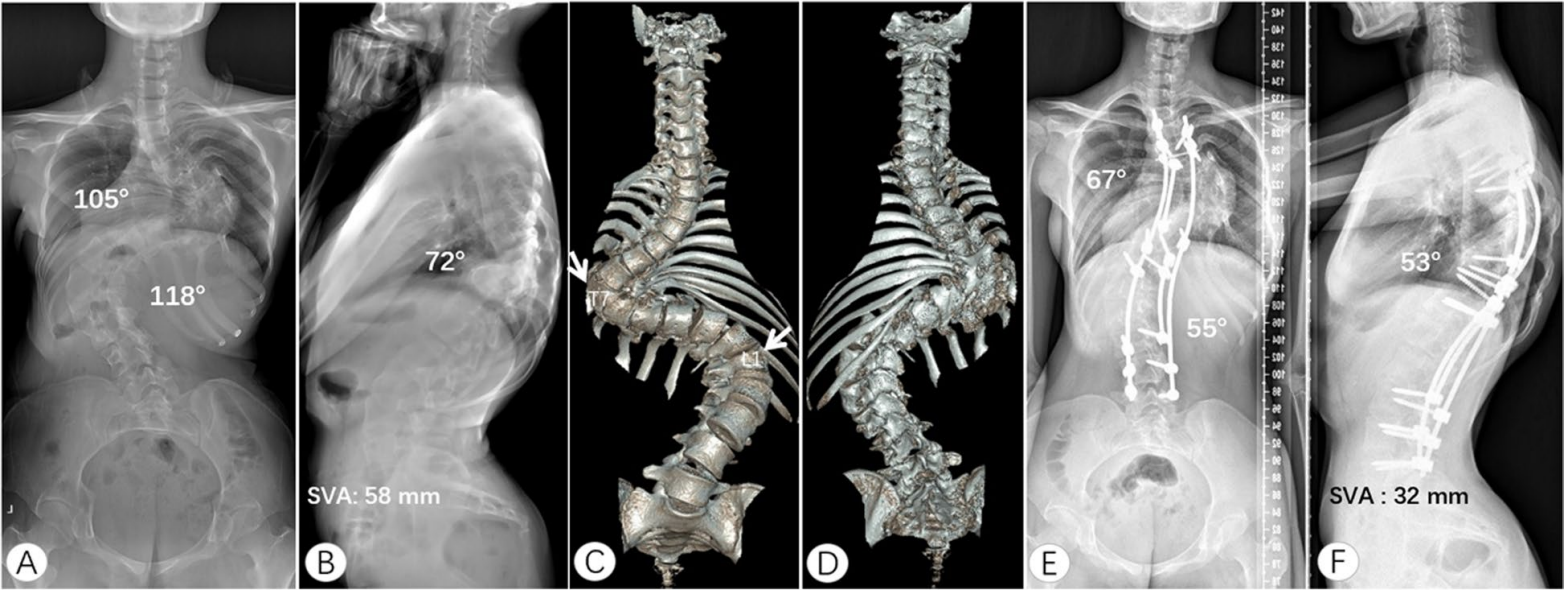
An 18-year-old female patient with rigid congenital kyphoscoliosis. The preoperative thoracic curve, lumbar curve and thoracic kyphosis were 105°, 118° and 74°, respectively. The sagittal plane was imbalance (**A**, **B**). Three-dimensional CT showed that the patient had T7 hemivertebra and posterior lamina deformity (**C**, **D**). She underwent posterior correction from T3 to L5. Short rods were placed on the apical regions of the thoracic curve and lumbar curve. After a 3-year follow-up, thoracic curve, lumbar curve, and kyphosis were improved to 67°, 55°, and 53°, respectively. The sagittal plane restored balance (**E**, **F**). SVA, sagittal vertical axis

**Fig. 3:**
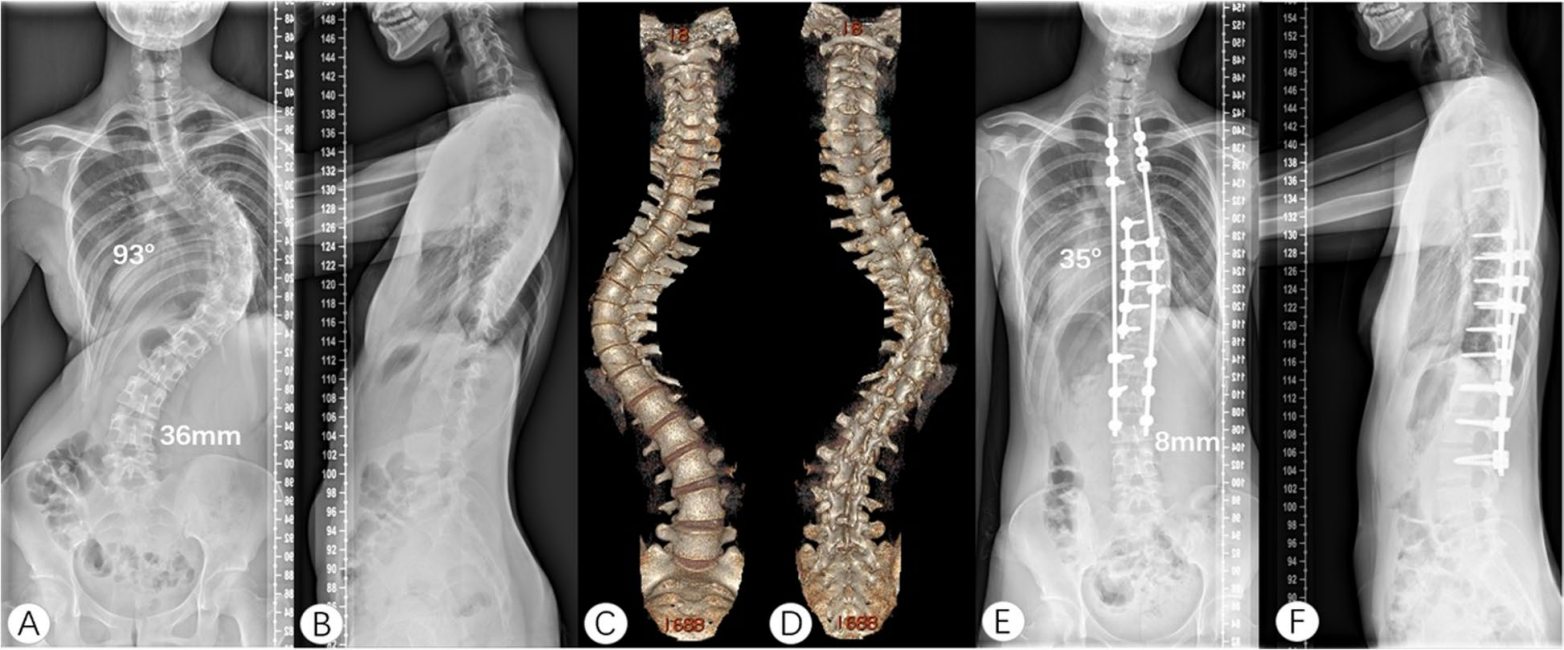
A 15-year-old female with idiopathic severe scoliosis (Lenke 1AN). The preoperative Cobb angle of the main curve was 93° and TS was 36 mm (**A**, **B**). The last touching vertebra of CSVL on her spine 3D reconstruction was L3 (**C**, **D**). She underwent posterior surgery from T3-L3. After three years of follow-up, the correction rate was 62.4% and TS was 8 mm (**E**, **F**). The correction remained stable and the coronal balance was improved. CSVL, center sacral vertical line. TS, trunk shift

The preoperative main curve of 101.6 ± 12.1° with a flexibility of 16.0 ± 9.9% was corrected to 48.6 ± 17.8° postoperatively, showing a 53.1% correction rate. At the final follow-up, the average main curve was 49.7 ± 14.9°, showing a 51.1% correction rate compared with the preoperative value and only a 2.3% loss of correction compared with the postoperative value. The TS values were 28.3 ± 14.3 mm, 18.6 ± 13.7 mm, and 17.8 ± 10.2 mm before the operation, after the operation, and at the final follow-up, respectively. 26 patients showed coronal imbalance (TS > 20 mm) before the operation, and 23 of them showed significant improvement at the final follow-up (TS < 20 mm). The SVA was 35.2 ± 22.3 mm before the operation, 24.8 ± 14.7 mm after the operation, and 26.1 ± 13.5 mm at the final follow-up. 16 patients showed sagittal imbalance (SVA > 40 mm) before the operation, and 14 of them restored sagittal balance (SVA < 40 mm) at the final follow-up. The preoperative height was 154.8 ± 8.6 cm, whereas the postoperative height was 162.5 ± 13.4 cm. There were 14 patients with normal kyphosis before surgery, and 28 patients with normal kyphosis at the last follow-up. The Cobb angle, AVT, TK, LL, and height were significantly improved both immediately after the operation and at the final follow-up; details are listed in Table 2. Representative cases with images obtained before the operation, after the operation, and at the final follow-up are presented in Figs. 1, 2, 3.

**Table 2:** Radiological outcomes

Radiographic	Preoperative	Postoperative	CR^a^(%)	*P*-value^b^	Follow-up	CR^c^(%)	*P* value^d^
**Coronal plane**							
**Main curve (°)**	101.6 ± 12.1	48.6 ± 17.8	53.1	0.000*	49.7 ± 14.9	51.1	0.000*
**AVT of the main curve (mm)**	103.3 ± 17.6	64.2 ± 16.4	/	0.000*	67.4 ± 19.8	/	0.000*
**Compensatory curve (°)**	61.1 ± 15.7	39.2 ± 19.5	35.8	0.000*	39.8 ± 19.7	34.9	0.000*
**AVT of the compensatory curve (mm)**	18.2 ± 12.1	17.4 ± 12.2	/	0.014*	16.5 ± 15.1	/	0.001*
**Trunk shift (mm)**	28.3 ± 14.3	18.6 ± 13.7	/	0.28	17.8 ± 10.2	/	0.91
**Sagittal plane**							
**Thoracic kyphosis (°)**	58.5 ± 18.6	42.4 ± 13.9	27.5	0.002*	43.9 ± 12.7	24.8	0.003*
Hypokyphosis (*n* = 1)	17.8	32.2			30.6		
Normal kyphosis (*n* = 14)	45.1 ± 2.9	39.1 ± 5.9		0.016*	38.9 ± 4.2		0.012*
Hyperkyphosis (*n* = 26)	67.3 ± 10.1	44.6 ± 11.8		0.000*	47.1 ± 12.7		0.000*
**Lumbar lordosis (°)**	62.7 ± 17.2	57.2 ± 12.5	/	0.021*	57.8 ± 10.8	/	0.029*
**Pelvic tilt (°)**	14.1 ± 12.5	13.9 ± 11.1	/	0.35	12.6 ± 9.8	/	0.77
**Pelvic incidence (°)**	42.4 ± 14.7	41.9 ± 14.1	/	0.06	40.7 ± 12.8	/	0.45
**Sacral slope (°)**	32.8 ± 11.3	35.4 ± 8.6	/	0.22	34.9 ± 7.9	/	0.30
**Sagittal vertical axis (mm)**	35.2 ± 22.3	24.8 ± 14.7	/	0.23	26.1 ± 13.5	/	0.12

CR^a^ indicated the correction rates before the operation and after the operation; *P*-value^b^ means comparison before the operation and after the operation

CR^c^ means correction rates before the operation and at the final follow-up; *P* value^d^ means comparison between before the operation and at the final follow-up

^*^ Shows significant difference; AVT, apex vertebra translation

### Postoperative complications

No complications occurred in any of the patients after surgery or during the follow-up period, including nervous system injury, pulmonary complications, wound infection, implant failure, or pseudoarthrosis.

### SRS-22 outcome data

Compared with the scores before the operation, significant improvement in the SRS-22 scores was noted at the final follow-up in the domains of SRS global score, pain, self-image, mental health, and satisfaction with the treatment (Table 3).

**Table 3:** SRS-22 data

	**Preoperative**	**Final follow-up**	***P*-value**
SRS Global score	3.5 ± 0.3	4.0 ± 0.3	0.001*
Function	2.8 ± 0.3	2.8 ± 0.4	0.17
Pain	2.6 ± 0.3	3.5 ± 0.3	0.002*
Self-image	2.4 ± 0.5	3.6 ± 0.4	0.000*
Mental health	3.2 ± 0.5	3.9 ± 0.4	0.000*
Satisfaction	2.3 ± 0.6	4.0 ± 0.3	0.000*

SRS-22, Scoliosis Research Society 22-item questionnaire

^*^ Shows significant difference

## Discussion

In this study, the surgical strategy of apical region correction and global balance with 3 rods provided reasonable corrections, good coronal and sagittal balance, significant improvement in the SRS-22 scores, and lower incidence of complications and implant failure. It could be a safe and effective optional surgical method for the treatment of severe and rigid scoliosis.

Schwab grade III–VI osteotomy such as PSO and VCR are powerful techniques to correct severe and rigid scoliosis. Lenke et al. reported a correction rate of 67% in 37 patients with severe and rigid scoliosis treated with VCR [22]. Other studies have reported that the correction rate of VCR in the treatment of severe scoliosis is 51%–59% [27]. However, the incidence of neurological complications and implant failure is high. Lenke et al. investigated the complications in 147 patients with severe spinal deformities who underwent VCR and found that 86 (59%) had intra- and postoperative complications, whereas 39 (27%) had neurological complications during the operation [22]. Bao et al. used the sequential correction technique to perform asymmetrical three-column osteotomy in the apical region of adult spinal deformity and used a short rod on the convex side to close the osteotomy area. This approach achieved satisfactory results but 5% of the patients experienced rod breakage [28]. Other studies have reported an incidence of perioperative complications of VCR of 32%–40% [20, 29]. Besides, high grade osteotomy of the thoracic vertebrae will shorten the thoracic height and reduce the thoracic volume in disguise, which may have a negative effect on patients' pulmonary function.

Patients with severe and rigid scoliosis generally have relatively severe thoracic deformities, often complicated by cardiopulmonary insufficiency. In our study, 95% of patients had moderate to severe restrictive or mixed ventilatory dysfunction, and 65% of patients had mild to moderate malnutrition (BMI < 18.5 kg/m^2^), with an average BMI of 17.8 kg/m^2^. These patients may not tolerate VCR due to the long operation time and massive bleeding, nor can they bear the risk of pulmonary complications caused by anterior surgery. For these patients, preoperative traction may be a good choice, which can improve both pulmonary function and nutritional condition in some patients, but the treatment cycle of traction is long and many patients find it intolerable.

With the invention and improvement of pedicle screws, posterior correction and fusion alone can achieve good results in the treatment of severe and rigid scoliosis, but the incidence of implant failure and pseudoarthrosis was 15% to 27%, which cannot be ignored [30, 31]. Owing to a stiff spine, some patients’ coronal or sagittal balance is difficult to restore and maintain. Li et al. used the sequential correction technique and transpedicular duet screws to treat severe thoracic idiopathic scoliosis. However, the transpedicular duet screws made the long rod on the concave side usually need to pre-bent to a larger angle on the coronal plane to connect with the short rod, which affected the restoration of the global balance [32]. In our study for severe and rigid scoliosis (major curves > 90°), the surgical strategy of apical region correction and global balance with 3 rods provided major curve correction of 53%. As for the spinal balance, 88% of the patients (23/26) with preoperative coronal imbalance (TS > 20 mm) restored coronal balance at the final follow-up and 87% of the patients (14/16) with preoperative sagittal imbalance (SVA > 40 mm) restored sagittal balance at the final follow-up. Both coronal and sagittal balance was improved after surgery and was maintained for at least 2 years. The average blood loss was 941 mL, with an average operation time of 286 min. Compared with VCR or anterior release and posterior spinal fusion in the treatment of severe spinal deformities, apical region correction and global balance with 3 rods showed shorter operation time and less blood loss [22]. Since only partially or fully resect the bilateral facet joints, the height of the chest is increased, which may improve patients’ pulmonary function [33]. As for LIV, we chose the last touching vertebra of the CSVL on the 3D reconstruction of the spine CT as the LIV to maximize motion segments. In a few cases, if the LIV was not leveled enough during the operation, we would lengthen one vertebra downward. This may be different from the traditional view of using stable vertebra or neutral vertebrae as LIV [34]. But Kim DH et al. recently found that the last touching vertebra on supine radiographs can be the optimal lower instrumented vertebra in adolescent idiopathic scoliosis patients [35]. During the follow-up of our patients with severe and rigid scoliosis, there was no severe adding on. The SRS-22 questionnaire revealed significant improvement in the scores for pain, self-image, mental health, and satisfaction with the treatment domains. Patients included in this study had no neurological complications, pulmonary complications or wound infections. During the follow-up, there was no failure of internal fixation or pseudoarthrosis. The incidence of complications was much lower than that of traction, VCR and anterior release followed by posterior spinal fusion.

From these results and our opinion, the reasonable corrective effect and low complication rate of apical region correction and global balance with 3 rods in the treatment of severe and rigid scoliosis mainly depend on the following. First, the short rod on the concave side can provide a strong stretching force. The apical region is the stiffest region of scoliosis. The short rod on the concave side only needs to be pre-bent to a small degree, which makes the load line close to its physical shape. Through the rod-rotation and distraction, it can provide stronger support in the apical region, thus improving the corrective effect and stability. Second, the apical region correction can reduce the pre-bending degree of the bilateral long rod and indirectly improve the global balance. After the short rod is rotated and distracted in the concave apical region, the stiffest area of scoliosis has been partially corrected and the Cobb angle of the main cure could be directly reduced, and the long rod, which was responsible for restoring the coronal balance, was applied more friendly. It can greatly avoid the pulling out of pedicle screws when using translation technique. Moreover, a smaller angle of pre-bending long rods will help the sagittal plane close to the expected design; therefore, the sagittal plane reconstruction can be simplified. Third, the triple rods and crosslinks can disperse the stress among the internal fixation instruments and maintain the rotation of the vertebrae, thus reducing the incidence of implant failure. Fourth, Schwab grade I or II osteotomy within the entire fusion range can reduce the incidence of neurological complications and create good condition for bone graft fusion to avoid pseudoarthrosis. The average BMI of the patients in this study was low (17.8 kg/m^2^), which was also beneficial to the stability of internal fixation. Fifth, one-stage posterior surgery can reduce the operation time, hospital stay and incidence of pulmonary complications.

The limitations of this study mainly lie in the small number of cases and heterogeneity of etiology. Nonetheless, the same senior surgeon operated on all patients. Further study is needed to explore the characteristics of surgical treatment of patients with different etiology. In addition, the study was limited by all the shortcomings associated with a retrospective study.

## Conclusion

The apical region correction and global balance with 3 rods surgical strategy could effectively correct severe and rigid scoliosis. The incidence of complications of neurologic injury, pulmonary injury and internal fixation was low. It may be safer for patients with malnutrition or poor pulmonary function.

## Data Availability

The dataset supporting the conclusions of this article is available from the corresponding author on reasonable request. Administrative permission was received from the Peking Union Medical College Hospital to access the medical records.

## Abbreviations

CD: Cotrel-Dubousset
CSVL: Center sacral vertical line
LIV: Lowest instrumented vertebra
3D: Three-dimensional
AVT: Apical vertebral translation
LL: Lumbar lordosis
PSO: Pedicle subtraction osteotomy
SVA: Sagittal vertical axis
SRS-22: Scoliosis Research Society 22-item
TK: Thoracic kyphosis
TS: Trunk shift
VCR: Vertebral column resection
